# First Tests of ^6^*Li* Doped Glass Scintillators for Ultracold Neutron Detection

**DOI:** 10.6028/jres.110.040

**Published:** 2005-06-01

**Authors:** G. Ban, X. Fléchard, M. Labalme, T. Lefort, E. Liénard, O. Naviliat-Cuncic, P. Fierlinger, K. Kirch, K. Bodek, P. Geltenbort

**Affiliations:** Laboratoire de Physique Corpusculaire, CNRS(IN2P3)-ENSI, F-14050 Caen Cedex, France; Paul Scherrer Institute, CH-5232 Villigen PSI, Switzerland; Institute of Physics, Jegellonian University, PL-30059 Cracow, Poland; Institut Laue-Langevin, F-38042 Grenoble Cedex 9, France

**Keywords:** glass scintillators, UCN detectors

## Abstract

We report the results of test measurements aimed at determining the performances of ^6^Li doped glass scintillators for the detection of ultra-cold neutrons. Four types of scintillators, GS1, GS3, GS10 and GS20, which differ by their ^6^Li concentrations, have been tested. The signal to background separation is fully acceptable. The relative detection efficiencies have been determined as a function of the neutron velocity. We find that GS10 has a higher efficiency than the others for the detection of neutrons with velocities below 7 m/s. Two pieces of scintillators have been irradiated with a high flux of cold neutrons to test the radiation hardness of the glasses. No reduction in the pulse height has been observed up to an absorbed neutron dose of 1 × 10^13^ cm^−3^.

## 1. Introduction

The development of detectors for cold and ultracold neutrons (UCN) has often resulted from the adaption of techniques currently used in the detection of thermal neutrons. The general principle of these techniques [1] combines the presence of elements having a large neutron absorption cross section, like ^3^He, ^6^Li and ^10^B, with an adapted detection technique for the secondary light fission fragments.

During the last couple of decades, the standard counter for cold and UCN has been the ^3^He or BF_3_ filled gas detector in which the absorbing element is part of the charge multiplication medium. The development of new UCN sources, with significantly increased intensities, has motivated the development of new detection techniques [2] which best fulfill the requirements of dedicated experiments planned near these sources.

Glass scintillators doped with ^6^Li are known to be a simple, robust and efficient detection tool for thermal neutrons. Their use for UCN has not been proven experimentally prior to this work.

## 2. Context and Specifications

### 2.1 Motivation

The present tests were carried out in order to compare the performances between glass scintillators and other UCN detectors. The most suitable counter will be used for a new neutron EDM measurement at PSI. In this context the UCN counters should fulfill a number of criteria associated with the experimental conditions. The most relevant requirements are: *i*) the detectors must have a high detection efficiency over the UCN velocity range between 4 m/s to 8 m/s. The comparison of efficiency figures should take into account the losses due to dead layers, like windows or light reflectors, as well as those due to critical velocities of active materials and edge effects. It should also include losses due to electronic conditions and cuts; ii) the time response must be sufficiently fast to cope with neutron counting rates up to 10^5^ s^−1^ cm^−2^. This is of importance when running the experiment near the new UCN source, SUNS, at PSI; iii) the gain of the system has to be stable or controlled under varying rates like those associated with the counting of UCN during emptying the chambers used for the EDM measurement; iv) the system must have a low sensitivity to background radiation, most often *γ*-rays, or be able to discriminate in a simple way between neutrons and gammas; v) the isotopes used for the neutron absorption have to be well packed to avoid diffusions or leaks which could contaminate the neutron guides and other parts of the setup, resulting in neutron losses; vi) when active detection elements are exposed directly to the neutrons, they must be radiation hard to avoid gain losses under moderate neutrons doses of about 10^8^ cm^−2^ year^−1^. vii) as a general rule it is suitable that the whole detection system, including the associated electronics, be simple to operate and maintain and that the cost be adapted to the performances.

### 2.2 Properties of Glass Scintillators

Glass scintillators are robust Ce doped glasses which can operate over a very wide range of temperatures, from −200 °C to 250 °C [3]. Those designated by “GS*x*” contain Ce_2_O_3_, SiO_2_, MgO, Al_2_O_3_ and Li_2_O oxides. Their densities are of the order 2.42 g/cm^3^ to 2.66 g/cm^3^ and their refractive index of 1.55–1.58. The peak emission for light is at 395 nm what allows to use a wide variety of photomultipliers without special wavelength shifters. They are very fast, with typical fall times of 60 ns and transparent to their own light for thicknesses smaller that 3 mm. The main difference between the GS types is the ^6^Li content. The properties of the four types used in these tests are presented in Table 1.

The first column indicates the scintillator type, the second lists the percentage in weight associated to the fraction of the Li_2_O oxide which contains the ^6^Li isotope, the third is the corresponding density, *ρ*, of ^6^Li atoms and the last is the critical velocity, *v*_c_. This velocity has been calculated using the chemical composition of the glass scintillators [3] and tabulated data of the neutron scattering lengths [4].

The neutron detection is performed by the capture reaction

$$n{+}^{6}\text{Li}\to t\;+\;\alpha$$ (1)

The energy of the triton (2.73 MeV) and that of the *α* particle (2.05 MeV) are fully deposited in the scintillator except for events where the capture takes place near the edges. Due to “quenching” effects and inhomogeneities in the glass composition and doping, the typical energy resolutions obtained with thermal neutrons on the full energy peak are of the order of 15 % to 20 % (FWHM) [3].

## 3. Test Measurements

The measurements described here have been performed with UCN from the PF2 facility at the Institut Laue-Langevin in Grenoble.

### 3.1 The Setup

The setup consisted of three main parts: a chopper made of several rotating wheels, a cylindrical UCN guide and a small detector chamber. All the elements of the setup were disposed at the same vertical level than the exit from the UCN turbine. The chopper is used to determine the UCN velocities. The detectors were located inside the small chamber. The distance from the chopper opening to the front of the scintillator was 83 cm. A plastic collimator with Ø18 mm was located at 20 mm from the scintillator front edge.

### 3.2 The Scintillators

Four types of glass scintillators, from Applied Scintillator Technologies^1^ have been tested. They were mounted in three pieces: one of GS1 (500 *µ*m thick), another of GS10 (250 *µ*m thick) and the third consisting of two scintillators GS20/GS3 (2 × 250 *µ*m thick) glued together. This last piece was used to better observe possible edge effects due to different ^6^Li dopings. The scintillator pieces were rectangular in shape, with a size 60 mm^2^ × 20 mm^2^. The scintillators were coupled to a photomultiplier tube (PMT) Photonis^1^ PM1911 through a Plexiglas light guide. For the GS1 and GS10 pieces, a 200 nm Al reflector layer was evaporated on the opposite face to the Plexiglas. This is the only dead layer traversed by the UCN before entering the scintillators. For the GS20/GS3 piece an Al foil reflector, 13 *µ*m thick, was used in order to be able to remove it. Under typical running conditions the chopper duty cycle was about 6 %.

### 3.3 Signals and Electronics

The signals from the PMT were amplified and integrated in a standard way. Three informations have been recorded for each event: the total charge of the signal from the PMT, *Q*_T_, the charge of the slow component of the signal, *Q*_F_, and the time-of-flight (TOF) relative to a signal associated with the opening of the chopper. The charge of the slow component was recorded for eventual discrimination of background. The TOF measurement was used to study the detector response as a function of the UCN velocity. It also allowed to estimate the uncorrelated background which is not discriminated by the pulse height.

### 3.4 Raw Data

**Fig. 1:**
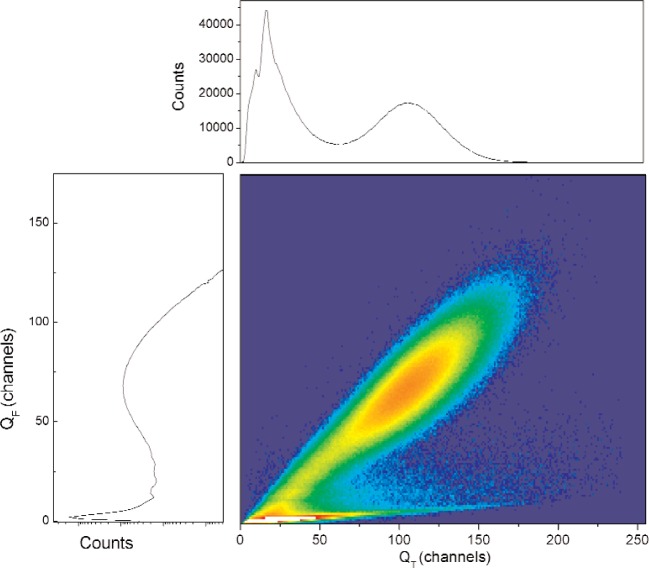
Two dimensional distribution between the amplitude of the slow component, *Q*_F_, and the total charge, *Q*_T_. The projected spectra are shown on the top and on the side.

**Fig. 2:**
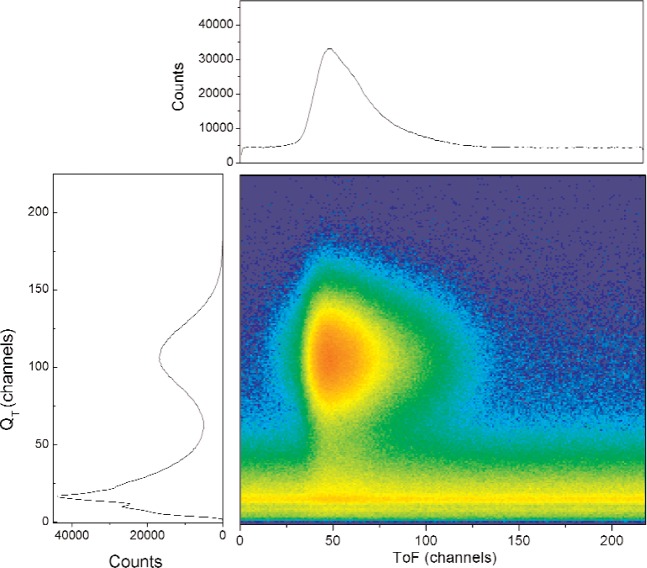
Two dimensional distribution between the TOF and the total charge *Q*_T_ from the PMT. The projected spectra are shown on the top and on the side.

The on-line data for the GS10 scintillator are shown in Figs. 1 and 2. The main distribution in the center of Fig. 1 is associated to the UCN as can be seen from the correlation with the TOF measurement in Fig. 2. The events in Fig. 1 having a smaller *Q*_F_ distribution but similar total charge are attributed to Cerenkov events in the Plexiglas which had a relatively large volume.

## 4. Results

**Fig. 3:**
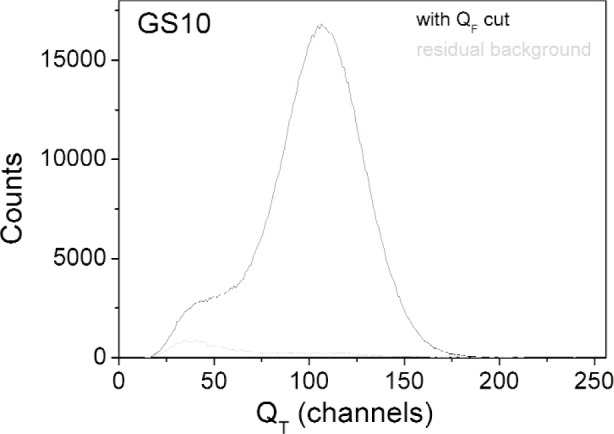
Total charge distribution and associated accidental background for the GS10 scintillator.

For each measurement the events associated with small amplitudes of the slow component have been cut from the amplitude spectra using the two dimensional distributions like the one shown in Fig. 1. The random events have been subtracted using events with large TOF. Figure 3 shows, for GS10, the spectrum of the total charge after the cuts and the corresponding uncorrelated background.

**Fig. 4:**
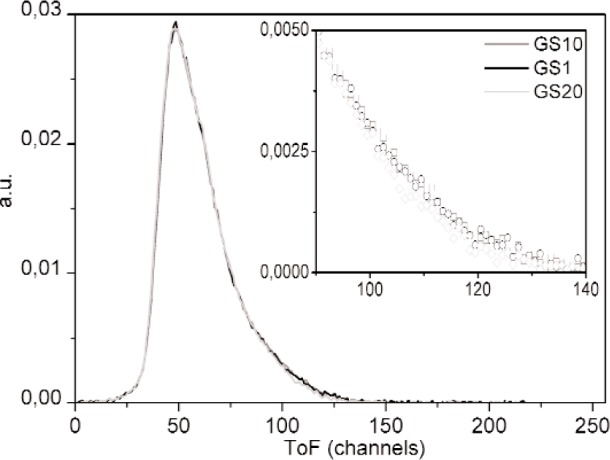
Time of flight distributions after subtraction of accidental events, normalized to the total number of counts.

**Fig. 5:**
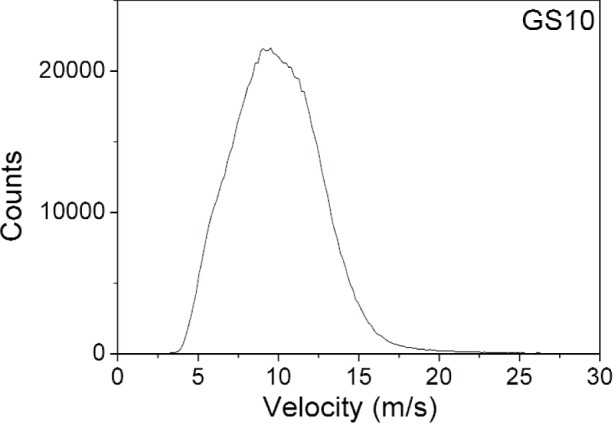
Velocity distribution of UCN from the PF2 source, deduced from the TOF measurement with a GS10 glass scintillator.

**Fig. 6:**
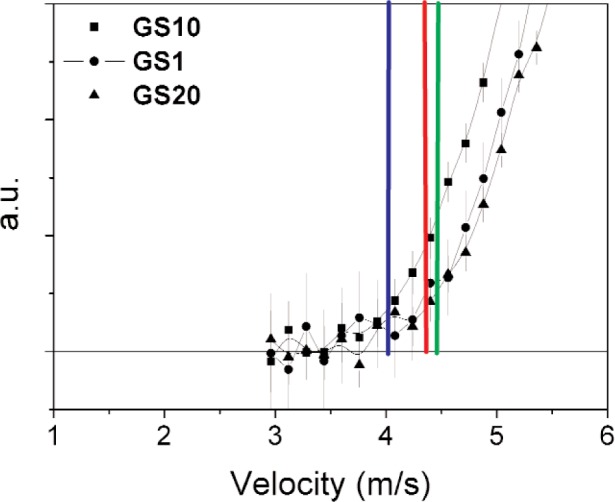
Lower edge of the velocity distributions obtained with GS10, GS1 and GS20 glass scintillators. The vertical lines indicate the calculated critical velocities.

The TOF distributions after accidental subtraction are shown in Fig. 4 for GS10, GS1 and GS20. The corresponding velocity distribution for GS10 is shown in Fig. 5. The lower part of the velocity distributions for the same three scintillators is shown in Fig. 6. It is seen that there are systematic differences on the low velocity edges of the distributions. These differences follow the associated critical velocities of the scintillators indicated by the vertical lines.

**Fig. 7:**
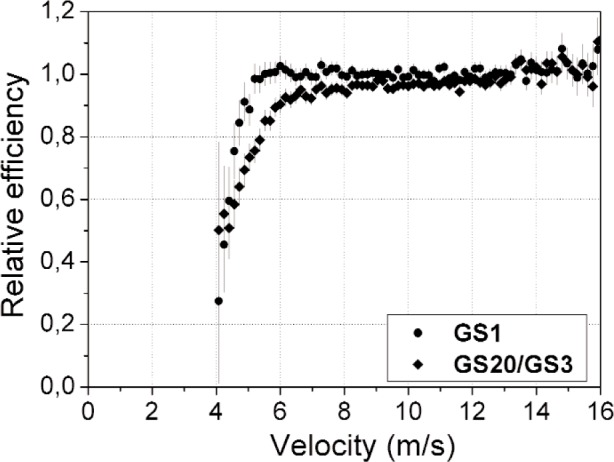
Efficiencies of GS1 and GS20/GS3 relative to GS10. See text for details.

The relative efficiency of the scintillators have been compared as a function of the UCN velocity. To this end the number of counts in the charge distribution of the GS1 and GS20/GS3 spectra have been divided by the number of counts of the GS10 spectrum, for several bins of the velocity distribution. These ratios have then been normalized to the upper part of the velocity spectrum, above 16 m/s, where it is assumed that all efficiencies are identical. The results are presented in Fig. 7 and show that GS10 has a higher efficiency than GS1 toward lower velocities. Although the edge effects are expected to be larger for GS10 due to the higher ^6^Li density it appears that the efficiency figures are dominated by losses due to the critical velocity. The comparison with GS20/GS3 is less straightforward as the experimental conditions were not identical.

## 5. Radiation Hardness Test

A test measurement has been carried at the SINQ source at PSI with the aim to observe eventual modifications of the optical properties of the scintillators under large neutron doses.

Two pieces of GS20 glass scintillator, 1 mm thick, with a surface of 20 mm^2^ × 20 mm^2^ have been irradiated by a high flux of cold neutrons at the FUNSPIN area. The estimated absorbed neutron doses were respectively 3 × 10^9^ cm^−3^ and 1 × 10^13^ cm^−3^. The neutron flux has also been monitored by activation of Au foils.

For each piece the amplitudes of the signals have been measured before and after irradiation. No measurable effect has been observed within the level of reproducibility of the detector test bench. This indicates that glass scintillators provide a robust solution with respect to aging effects due to neutron irradiation.

## 6. Improvements

The widths of the charge distributions presented above are larger than expected for these scintillators. This was due to a non optimal light collection during these tests. Measurements with very cold neutrons and tests performed in the laboratory with moderated neutrons from radioactive sources showed that typical energy resolutions in the range 15 % to 20 % FWHM can indeed be achieved.

## 7. Conclusions

We have shown in this work that ^6^Li doped glass scintillators are adapted to detect UCN. Among the four different tested types, GS10 has a higher efficiency than GS1 and GS20 for UCN velocities smaller than 7 m/s. This follows the comparison of the estimated critical velocities. The highest ^6^Li density of the GS10 scintillator allows to use thinner layers while keeping the edge effects at a tolerable level. Two samples of GS20 scintillator have been irradiated with cold neutrons. No measurable effect has been observed up to an absorbed dose of 1 × 10^13^ n/cm^3^.

## Figures and Tables

**Table 1:** Physical data of some GS*x* glass scintillators

GS type	^6^Li_2_O (%)	*ρ*(^6^Li) (cm^−3^)	*v*_c_ (m/s)
GS3	∼0	6.4 × 10^17^	4.4
GS1	0.45	5.1 × 10^20^	4.4
GS10	1.35	1.8 × 10^21^	4.0
GS20	17.0	2.2 × 10^22^	4.5

## Footnotes

1 Certain commercial equipment, instruments, or materials are identified in this paper to foster understanding. Such identification does not imply recommendation or endorsement by the National Institute of Standards and Technology, nor does it imply that the materials or equipment identified are necessary the best available for the purpose.
